# Parental and adolescent opinion on COVID-19 vaccination among 12-17-year-olds: A hospital-based study

**DOI:** 10.6026/973206300213832

**Published:** 2025-10-31

**Authors:** Akash Yadav, Sardar Vikram Singh Bais, Ambuj Kumar Soni, Ankit Jain

**Affiliations:** 1 Department of Pediatrics, Shrimant Rajmata Vijayaraje Scindhiya Medical College, Shivpuri, Madhya Pradesh, India; 2 Department of Pediatrics, Birsa Munda Govermemt Medical College, Shahdol, Madhya Pradesh, India; 3 Department of General Surgery, Birsa Munda Government Medical College, Shahdol, Madhya Pradesh, India; 4 Department of Pediatrics, Shyam Shah Medical College and Associated Gandhi Memorial hospital, Sagar, Madhya Pradesh, India

**Keywords:** Covid 19, vaccination, patents intention

## Abstract

Vaccine hesitancy among parents and adolescents poses a major barrier to achieving optimal COVID-19 vaccination coverage. This hospital- based study at Gandhi Memorial Hospital, Rewa, included 1500 parents and 1695 adolescents aged 12-17 years to assess vaccination intentions and influencing factors. Overall, 63% of parents and 28% of adolescents expressed willingness to vaccinate, with fear of side effects being the predominant concern (> 90%). Parental willingness was significantly associated with younger age, female gender, higher education and prior vaccination (p < 0.001). Strengthening awareness and addressing safety concerns are essential to improve vaccine acceptance among adolescents.

## Background:

The emergence of severe acute respiratory syndrome coronavirus 2 (SARS-CoV-2) in Wuhan, China, in late 2019 led to a global pandemic with unprecedented health, social and economic consequences [1]. As of January 2024, over 702 million confirmed cases and 6.9 million deaths have been reported worldwide [2]. In India, more than 45 million cases and 533,000 deaths have been documented [3]. While adults have been disproportionately affected by severe COVID-19 outcomes, children and adolescents are not immune to infection and can contribute to community transmission [4]. Children with COVID-19 typically exhibit milder symptoms and are less likely to require hospitalization or face fatal outcomes compared to adults [5]. However, they can still experience significant morbidity and contribute to viral spread within communities [6]. The role of children in transmission has gained attention as countries consider reopening schools and relaxing social distancing measures [7]. In September 2021, children accounted for one-fourth of all COVID-19 cases in India, highlighting their potential role in disease dynamics [8]. Vaccination has emerged as a critical strategy to control the COVID-19 pandemic, with multiple vaccines demonstrating safety and efficacy in various populations [9]. The World Health Organization (WHO) and the US Centers for Disease Control and Prevention recommend COVID-19 vaccination for eligible age groups, including adolescents [10]. In India, the COVID-19 vaccination program began in January 2021 for adults, with subsequent expansion to adolescents aged 12-17 years [11]. Despite the availability of vaccines, vaccine hesitancy remains a significant barrier to achieving optimal vaccination coverage [12]. Previous studies have reported varying rates of parental willingness to vaccinate their children against COVID-19, ranging from 32.8% in the Democratic Republic of Congo to 89% in England [13]. In India, limited research has focused on parents' intentions and perceptions regarding COVID-19 vaccination for their children, particularly adolescents [14]. Understanding parental and adolescent intentions regarding COVID-19 vaccination is crucial for designing effective public health strategies and communication campaigns. Factors influencing vaccination decisions include concerns about vaccine safety, perceived risk of COVID-19, trust in healthcare systems and socio-demographic characteristics [15]. However, there remains a gap in understanding these factors specifically within the Indian context, where cultural, social and economic factors may uniquely influence vaccination decisions [16]. Therefore, it is of interest to measure parental intention to vaccinate their adolescent children (12-17 years) against COVID-19, as well as the adolescents' own vaccination intentions and identify associated socio-demographic factors in a tertiary care center in India.

## Materials and Methods:

## Study design and setting:

A hospital-based observational study was conducted at the Department of Pediatrics, Gandhi Memorial Hospital, associated with Shyam Shah Medical College, Rewa, Madhya Pradesh, India. The study period was from August 2022 to July 2023.

## Study population and sample size:

The study population included parents and their children aged 12 to 17 years visiting the outpatient department. The sample size was calculated based on a previous study in India by Sudharsanan *et al.*, which reported a 33.5% prevalence of parental intention to vaccinate children against COVID-19. Using the formula for sample size calculation with a prevalence of 33.5%, marginal error of 2.5% and 95% confidence level, the calculated sample size was 1359. Accounting for potential non-response, a final sample size of 1500 parents was targeted. A total of 1500 parents and their 1695 children were included in the study.

## Inclusion and exclusion criteria:

Parents with children aged 12-17 years who provided informed consent were included in the study. Children aged 12-17 years who provided assent were included. Those who refused to participate or had cognitive impairments preventing them from understanding the questionnaire were excluded.

## Study instrument:

A structured questionnaire was developed by a team of experts including a pediatrician and a public health specialist. The questionnaire consisted of pre-tested open-ended and closed-ended questions and was divided into sections:

[1] Socio-demographic information: age, gender, residence, education, occupation, socio-economic status (using the modified Kuppuswamy scale) and family type.

[2] COVID-19-related information: history of COVID-19 infection, vaccination status of parents and children, number of vaccine doses received and COVID-19-related deaths in the family.

[3] Perception and intention regarding COVID-19 vaccination: willingness to vaccinate, perceived benefits and risks, reasons for willingness or hesitation and sources of information.

The questionnaire was pre-tested on 50 participants to assess clarity and comprehension and necessary modifications were made before final administration.

## Data collection:

The principal investigator and trained research assistants conducted face-to-face interviews with parents and children in a private setting at the hospital. Participants were explained the purpose of the study in their preferred language and written informed consent was obtained from parents while assent was obtained from children. Data were collected regarding socio-demographic characteristics, COVID-19 experiences and vaccination intentions.

## Operational definitions:

[1] Literacy status: Defined according to guidelines provided by the Indian government's Department of School Education and Literacy. Participants were categorized as illiterate (unable to read or write in any language) or literate (with varying levels of formal education).

[2] Occupation: Categorized as unemployed, unskilled, semi-skilled, skilled, semi-professional, professional, or homemaker.

[3] Socio-economic status: Determined using the modified Kuppuswamy scale, incorporating education, occupation of the head of the family and monthly income, with categories ranging from upper class (class I) to lower class (class V).

[4] Family type: Classified as nuclear (husband, wife and unmarried children) or joint (two or more nuclear units staying together).

[5] Vaccine willingness: Parents were categorized as "willing" if they responded positively to vaccinating their children and "hesitant" if they responded negatively or were uncertain. Children were similarly categorized based on their responses.

## Statistical analysis:

Data were entered into Microsoft Excel and analyzed using Statistical Package for the Social Sciences (SPSS) version 26 and EPI-INFO version 7. Categorical variables were presented as frequencies and percentages, while continuous variables were expressed as mean ± standard deviation (SD). Chi-square tests were used to compare proportions between groups, with statistical significance defined as p < 0.05. Multivariable logistic regression analysis was performed to identify independent predictors of vaccination intention, with results presented as odds ratios (OR) and 95% confidence intervals (CI).

## Ethical considerations:

The study was approved by the Institutional Ethics Committee of Shyam Shah Medical College, Rewa (Approval No. SSMC/EC/2022/07). Written informed consent was obtained from all parents and assent was obtained from children aged 12-17 years. Participants were assured of confidentiality and data were stored securely to maintain privacy. The study adhered to the principles of the Declaration of Helsinki.

## Results:

Socio-demographic Characteristics of Parents The study included 1500 parents with a mean age of 49.88 ± 10.06 years. The majority were in the 41-50 years age group (43.7%), followed by 51-60 years (26.4%). Male parents predominated (63.5%) and most resided in urban areas (57.9%). Regarding education, 41.3% had secondary schooling, while 25.1% had primary education. In terms of occupation, 30.0% were skilled workers and 26.9% were homemakers. Most parents belonged to socio-economic class V (34.3%) and lived in nuclear families (65.3%) (Table 1 - see PDF). COVID-19 Profile and Vaccination Status of Parents Among the parents, 33.8% (n=507) reported a history of COVID-19 infection, while 66.2% (n=993) had no history of infection. The majority (70.9%, n=1064) had received at least one dose of COVID-19 vaccine, with 52.5% (n=788) having received two doses and 5.5% (n=83) having received a booster dose. Only 29.1% (n=436) were unvaccinated. Among the parents whose children had received the COVID-19 vaccine, 51.2% reported a very negative experience, while only 13.7% reported a very positive experience. COVID-19-related deaths in the family were reported by 1.3% (n=19) of parents. The primary source of information about COVID-19 vaccination was healthcare providers (57.6%), followed by news outlets (11.9%) (Table 2 - see PDF). Parental Perception and Intention Regarding COVID-19 Vaccination for Children Overall, 63.0% (n=945) of parents expressed willingness to vaccinate their children against COVID-19. The majority (73.9%) perceived COVID-19 as a serious illness and 58.9% believed that vaccinating adolescents is important for their health and the health of others. However, only 52.1% were aware of the potential benefits of the COVID-19 vaccine and 47.9% were aware of potential risks. Among parents unwilling to vaccinate their children (n=555), the primary reasons were concerns about side effects (92.1%), waiting for more information (44.9%) and personal or family decisions (31.9%). Among parents willing to vaccinate their children (n=945), the main reasons were the belief that the vaccine can prevent COVID-19 (89.0%), perception that the vaccine is safe (76.0%) and concern that their child is at higher risk of infection (63.0%) (Table 3 - see PDF).

Characteristics and Vaccination Status of Adolescents The study included 1695 adolescents with a mean age of 14.05 ± 1.39 years. The majority were in the 14-15 years age group (43.1%), followed by 12-13 years (37.8%). Female adolescents slightly predominated (50.2%). Regarding COVID-19 status, 15.8% (n=268) reported a history of infection, while 84.2% (n=1427) had no history of infection. Only 7.7% (n=131) of adolescents had received the COVID-19 vaccine, while the majority (92.3%, n=1564) were unvaccinated. Among vaccinated adolescents, 77.0% (n=101) reported experiencing side effects (Table 4 - see PDF). Adolescent Intention Regarding COVID-19 Vaccination Only 28.0% (n=474) of adolescents expressed willingness to receive the COVID-19 vaccine. Among those willing, the primary reasons were the belief that the vaccine can prevent COVID-19 (93.9%), perception that the vaccine is safe (82.7%) and concern about protecting others (67.7%). Among adolescents unwilling to be vaccinated (n=1221), the main reasons were concerns about side effects (94.0%), personal or family decisions (71.9%) and lack of information or awareness (48.9%) (Table 5 - see PDF). Factors Associated with Parental Intention to Vaccinate Children Parental age was significantly associated with vaccination intention, with willingness decreasing with increasing age (41-50 years: 77.4% vs. >60 years: 36.3%, p<0.001). Female parents were more willing to vaccinate their children compared to male parents (69.0% vs. 59.6%, p<0.001). Education level showed a complex relationship, with the highest willingness among graduates and above (78.0%) and illiterate parents (72.3%) and the lowest among those with primary education (46.3%, p<0.001). Socio-economic status also showed variation, with class V having higher willingness (64.7%) compared to class I (56.3%, p<0.001). Parents from nuclear families were more willing than those from joint families (68.6% vs. 52.5%, p<0.001). Parental vaccination status was strongly associated with intention, with unvaccinated parents showing lower willingness (26.1% vs. 84.6% for those with two doses, p<0.001). Parents with a history of COVID-19 infection were more willing to vaccinate their children (76.1% vs. 56.3%, p<0.001). COVID-19-related deaths in the family were associated with lower willingness (15.8% vs. 63.6%, p<0.001). Children's willingness was also associated with parental intention, with parents of willing children more likely to intend vaccination (68.8% vs. 60.3%, p<0.001) (Table 6 - see PDF). Multivariate Analysis of Factors Associated with Parental Intention Multivariate logistic regression analysis identified several independent predictors of parental intention to vaccinate their children. Parents under 40 years were more likely to intend vaccination (OR=3.48, 95% CI=1.80-4.75, p<0.001). Higher education was strongly associated with intention (OR=6.83, 95% CI=4.06-11.48, p<0.001). Upper socio-economic status (OR=3.54, 95% CI=2.20-5.70, p<0.001), joint family structure (OR=2.29, 95% CI=1.77-3.14, p=0.004) and personal COVID-19 vaccination (OR=2.30, 95% CI=1.17-3.53, p<0.001) were also significant predictors. Parents who had tested positive for COVID-19 were more likely to intend vaccination (OR=2.36, 95% CI=1.36-4.10, p=0.002). If the child was willing to be vaccinated, parents were significantly more likely to intend vaccination (OR=4.20, 95% CI=1.54-9.32, p<0.001) (Table 7 - see PDF).

## Discussion:

This study examined parental and adolescent intentions regarding COVID-19 vaccination and associated socio-demographic factors in a tertiary care center in India. The findings reveal that while a majority of parents (63.0%) are willing to vaccinate their adolescent children against COVID-19, only a minority of adolescents (28.0%) express willingness to be vaccinated themselves. Concerns about vaccine side effects emerge as the primary barrier to vaccination for both parents and adolescents. The parental willingness rate of 63.0% observed in our study is higher than that reported in a previous Indian study by Sudharsanan *et al.* (33.5%) [17] But lower than rates reported in countries like England (89%), New Zealand (80%) and China (73%) [18]. This variation may reflect differences in study timing, cultural contexts, healthcare systems and the stage of the pandemic when the studies were conducted. Our study was conducted during a period when COVID-19 cases had declined in India, potentially reducing the perceived urgency of vaccination. The finding that concerns about side effects (92.1%) were the primary reason for parental hesitancy aligns with previous studies conducted in various countries [19, 20]. This highlights the critical need for transparent communication about vaccine safety, potential side effects and their management. Healthcare providers, who were identified as the most trusted source of information (57.6%), play a crucial role in addressing these concerns and providing accurate information. Our study revealed that only 28.0% of adolescents were willing to be vaccinated, with concerns about side effects (94.0%) being the primary barrier. This low willingness rate among adolescents is concerning and suggests that vaccination campaigns targeting this age group need to address their specific concerns and involve them in decision-making processes. The finding that adolescents' willingness was significantly associated with parental intention underscores the importance of family dynamics in vaccination decisions. The association between parental age and vaccination intention, with younger parents (41-50 years) showing higher willingness (77.4%) compared to older parents (>60 years: 36.3%), is consistent with previous research [21]. This may reflect differences in health literacy, access to information, or risk perception across age groups. The finding that female parents were more willing to vaccinate their children (69.0% vs. 59.6%) contrasts with some previous studies [22] but aligns with others [23], suggesting that gender dynamics in vaccination decisions may vary across cultural contexts. The complex relationship between education level and vaccination intention observed in our study, with the highest willingness among graduates and above (78.0%) and illiterate parents (72.3%) and the lowest among those with primary education (46.3%), is intriguing. This non-linear pattern may reflect different information sources, trust levels, or risk perceptions across educational groups. Further research is needed to understand this relationship better. The strong association between parental vaccination status and intention to vaccinate children (unvaccinated: 26.1% vs. two doses: 84.6%) highlights the importance of parental modeling in vaccination decisions. This finding is consistent with previous research indicating that parents who are vaccinated themselves are more likely to vaccinate their children [24].

## Conclusion:

We show that while a majority of parents (63.0%) are willing to vaccinate their adolescent children against COVID-19, only a minority of adolescents (28.0%) express willingness to be vaccinated themselves. Concerns about side effects emerge as the primary barrier for both groups. Several socio-demographic factors, including parental age, gender, education, socio-economic status, family type and personal vaccination status, are significantly associated with parental intention to vaccinate their children.
